# Habitat Selection and Activity Patterns of Japanese Serows and Sika Deer with Currently Sympatric Distributions

**DOI:** 10.3390/ani11123398

**Published:** 2021-11-28

**Authors:** Yoshikazu Seki, Shin-ichi Hayama

**Affiliations:** 1 School of Veterinary Medicine, Nippon Veterinary and Life Science University, 1-7-1, Kyonancho, Musashino, Tokyo 180-8602, Japan; hayama@nvlu.ac.jp; 2 Department of Agri-Environmental Sciences, Tamagawa University, 6-1-1, Tamagawagakuen, Machida, Tokyo 194-8610, Japan

**Keywords:** *Capricornis crispus*, *Cervus nippon*, activity level, camera trap, habitat use, interference competition, niche, overlap, spatial partitioning, temporal partitioning

## Abstract

**Simple Summary:**

Investigating the interspecific interactions between species provides a suitable model for understanding the mechanisms of coexistence between sympatric species. We assessed the spatial and temporal partitioning of spaces between Japanese serows (*Capricornis crispus*) and sika deer (*Cervus nippon*), which are usually allopatric, in an area with early-stage invasion of sika deer into Japanese serow habitat. The habitat selection and activity patterns of the two species were evaluated using camera traps. Both species were recorded in >25% of the same camera sites and showed similar selection tendencies for water resources. The Japanese serows selected steep slopes, whereas the sika deer selected areas distant from human settlements, resulting in low spatial overlap. Additionally, the Japanese serows were more active during the daytime, whereas the sika deer were active at the crepuscule. The observed spatial and temporal partitioning likely reduces their encounter rates, thereby minimizing possible interference competition. However, spatial and temporal overlaps between the two species are likely to increase as the density of sika deer increases, potentially resulting in a decline in the density of Japanese serows with smaller body sizes. Trapping for deer management should be focused on areas with gentle slopes, away from valleys and human settlements, to reduce the unintentional capture of Japanese serows.

**Abstract:**

The Japanese serow (*Capricornis crispus*) and sika deer (*Cervus nippon*) in Japan are usually allopatric. However, a recent expansion in the distribution range of sika deer, combined with an increase in abundance, has resulted in an overlap of the distribution ranges of the two species. We examined the habitat selection and activity patterns of Japanese serows and sika deer using camera traps placed at 83 sites within a 210 km^2^ study area, where the distribution range of these two species has recently overlapped. Although both species were photographed throughout the study area, we observed a low spatial overlap between them. The Japanese serows selected steep slopes, and the sika deer selected areas far away from human settlements. In addition, the Japanese serows and sika deer exhibited temporal partitioning with diurnal and crepuscular activity patterns, respectively. The observed partitioning could be explained by differences in their species-specific habitat selections, rather than competition, because the photographic capture rate of the Japanese serows was not affected by that of the sika deer and vice versa. These partitioning behaviors are likely to reduce the rate of encounters between the two ungulates, which enables their coexistence considering the sika deer density remains low.

## 1. Introduction

Interspecific interactions influence species abundance and community composition [1]. Predation exerts significant influence on the dynamics of prey populations and their communities [2,3,4,5,6]. To reduce predation risk, herbivores may change their foraging behavior and habitat use [7,8,9,10]. Interspecific competition also influences species abundance, habitat use, and diet [11,12,13,14,15]. Ecologically similar sympatric species elude or reduce competition by spatial and temporal partitioning of habitat, or by exploiting different food resources, which may promote coexistence [16,17,18,19,20,21,22,23,24,25,26,27,28]. Studies on interspecific interactions are crucial for understanding the mechanisms of coexistence between sympatric species.

The distribution ranges of various deer species have expanded in recent decades worldwide, with drastic increases in abundance [29], thereby prompting more studies on the interspecific interactions between deer and other species; the modification of vegetation due to increased deer foraging has been well-documented [29,30,31]. By modifying vegetation, deer indirectly affect the population and community composition of small animals utilizing the vegetation as food resources or resting and breeding sites [29,32,33,34,35]. An increase in browsing pressure by deer can also lead to significant declines in the population of some large mammals through interspecific competition. For example, abundant white-tailed deer (*Odocoileus virgininianus*) caused an indirect extirpation of the black bear (*Ursus americanus*) population on Anticosti Island by over-browsing berry-producing shrubs [5]. Studies on interspecific competition among sympatric deer and other species are essential to identify ways of preventing such significant declines in wildlife populations.

Two ungulates, the sika deer (*Cervus nippon*) and the Japanese serow (*Capricornis crispus*), are normally allopatric in Japan [36,37]. However, recent population growth and range expansion in sika deer have resulted in an overlap in the distribution ranges of these two ungulates. Comparatively little is known about interspecific competition between Japanese serows and sika deer in regions where they occur sympatrically. However, declines in the population of Japanese serows, with increasing sika deer populations, have been reported in some areas in Japan [36,38]. This may be a result of interspecific competition between these two species. Although two comparative studies on the food habits of Japanese serows and sika deer exhibited differences in feeding selection between the two species living in the same area [39,40], their food habits were found to be similar in the area where the food supply was scarce due to heavy grazing by sika deer [41]. These results indicate that the extent of interspecific competition between the two ungulates could increase with the increasing population of sika deer.

However, even if there is a high dietary overlap between Japanese serows and sika deer in the same area, differences in their habitat use have been shown [42]. Takada et al. [43] also demonstrated that Japanese serows and sika deer have exhibited differences in their habitat use in terms of vegetation and topography. However, these two studies on habitat use were conducted at a relatively finer scale (1–2 km^2^) in areas where densities of sika deer are relatively high (10.4–15.0 individuals/km^2^). Since studies at different scales often yield a different numerical result or pattern [44], we also need to compare the habitat use between the two ungulates at a broader scale. In addition, in other areas with high sika deer densities (mean of 17.9 individuals/km^2^), the Japanese serows selectively chose habitats to avoid sika deer [37]. Since the spatial partitioning may be a result of previous competitive interactions and competitive exclusion [45,46,47], the Japanese serows may have shifted or might shift their ecological niche in the future in areas with relatively higher densities of sika deer. To adequately assess the interspecific competition between Japanese serows and sika deer, comparisons should be also made in areas with low sika deer densities. In addition to habitat and feeding-related drivers, time is a critical dimension in ecology [48]. Temporal partitioning may lead to a reduced competition where interspecific competition might otherwise occur [21,49]. Although the activity pattern of sika deer have been well documented [50,51,52,53,54], a systematic survey on the activity pattern of Japanese serow populations is lacking; only one study has included an individual radio-collared Japanese serow [55]. Therefore, whether Japanese serows and sika deer exhibit temporal partitioning in sympatric areas remains unclear.

On an evolutionary timescale, to minimize the loss of fitness incurred through competition, natural selection would be expected to promote clear partitioning in resource use between regularly interacting sets of species. Thus, evidence for actual competition between members of an established community of large herbivores is generally difficult to obtain [47]. Therefore, simultaneous studies on habitat use and activity between Japanese serows and sika deer, at different stages of range expansion of the sika deer, provides a model to comprehend the mechanisms of coexistence, not only for the two species but also for other ungulate communities. The assessment of the interspecific competition between Japanese serows and sika deer is also essential for the conservation of Japanese serows, which are solitary and territorial animals that typically live in low densities [56,57]. Although the Japanese serow has been legally protected since 1955, the species is listed as a threatened local population in four regions of Japan [58]. However, recently, many individual Japanese serows have been unintentionally captured in snare traps set for the management of sika deer, and approximately 30% of these captured individuals sustained injuries in the process [59]. Additional knowledge about the habitat selection behavior in sympatric populations of these two ungulates can enable effective management of sika deer and reduce the unintentional capture of Japanese serows.

The purpose of this study was to compare the habitat selection and activity patterns of sympatric Japanese serows and sika deer, using camera traps in an area with an early-stage invasion of sika deer into the habitat occupied by populations of Japanese serows at the landscape scale, i.e., the third-order habitat selection process defined by Johnson [60]. Since Japanese serows and sika deer are usually allopatric [36,37], we hypothesized that the two species may exhibit spatial partitioning in areas where their distribution has recently overlapped. Even if ungulates overlapped spatially, differences in activity patterns may reduce the risk of encounters [49,61]. Therefore, we also hypothesized that Japanese serows and sika deer may exhibit temporal partitioning in the sympatric area.

## 2. Materials and Methods

### 2.1. Study Area

**Figure 1:**
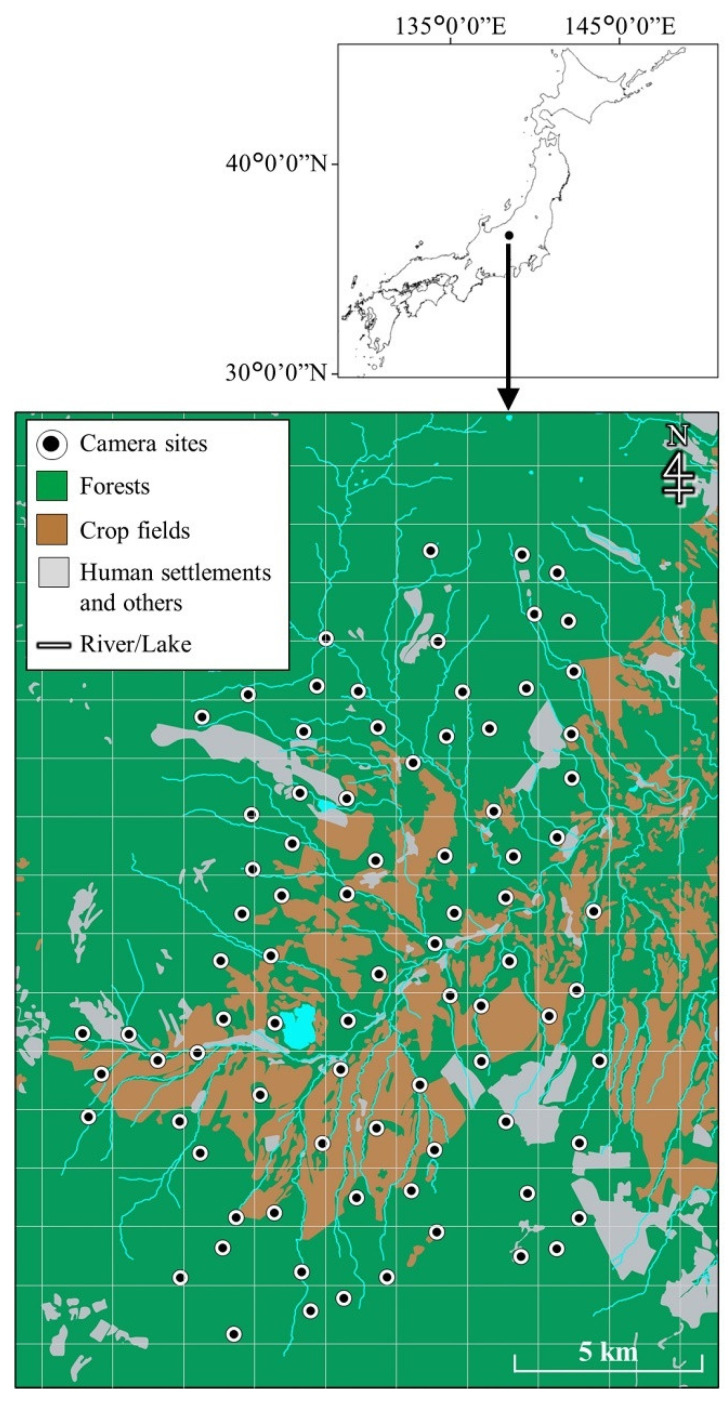
Study area in Tsumagoi, central Honshu, Japan. Camera traps were placed at 83 sites to monitor Japanese serows and sika deer between July and September 2012. The study area was divided into approximately 1.7 × 1.7 km grids. The map was created based on national land use information (Ministry of Land, Infrastructure, Transport and Tourism of Japan) and 6–7th Japanese National Survey of the Natural Environment (Ministry of the Environment of Japan).

The study was conducted in Tsumagoi Village (337.58 km^2^), Gunma Prefecture, central Honshu, Japan, which is located in a cool temperate zone (36°31.0′ N, 138°31.49′ E; 700–2550 m elevation; Figure 1). The study area is an important cabbage (*Brassica oleracea* var. *capitata*) growing area. Moreover, the tourism industry is important, and there are several leisure facilities, such as campgrounds, golf courses, cottage areas, hot-spring areas, and skiing areas. The vegetation of the study area is dominated by deciduous broadleaved trees and conifers. According to data collected from Tashiro Weather Station (1230 m), located in the southern part of Tsumagoi, between 1980 and 2010, the mean annual temperature was 7.1 °C (−4.6 °C in January and 19.5 °C in August) and the mean annual precipitation was 1506 mm. The ground was typically covered with snow from mid-December to mid-April.

The mean ± standard deviation (SD) of the population density estimated by the block count method [62], which was conducted in four areas in October 2012, was 1.77 ± 1.70–2.28 ± 2.66 for Japanese serows and 1.40 ± 0.95/km^2^ for sika deer. The Gunma Prefecture regarded Tsumagoi as a low density area for sika deer (<1.5 individuals/km^2^ in 2012, computed from Gunma [63]). Thus, we considered that the deer density was low during the study period. In Tsumagoi, the presence of sika deer was not observed during the 1996 survey, but the 2008 survey first confirmed the sparse distribution of sika deer [64]. Additionally, there were no reports of crop damage by sika deer before 2008; however, such reports have gradually increased since 2013 [65]. Therefore, we considered that the sika deer range has recently expanded into the study area, possibly between 1996 and 2008. In this area, almost no deer were observed by camera traps during the months between December and April in the period 2013–2015, possibly because they had migrated to other areas (Y Seki, unpublished data).

### 2.2. Camera Trapping

We divided the study area into approximately 1.7 × 1.7 km grids obtained by equally dividing the Secondary Area Partition (approximately 10 km^2^) into 36 units. We used 20 camera traps (Ltl-Acorn 5210A, Zhuhai Ltl Acorn Electronics Co., Ltd., Guangdong, China) to investigate the activity and habitat use patterns of Japanese serows and sika deer. Between July and September 2012, we placed camera traps at a total of 83 sites (one camera inside each grid cell) in the study area, covering an area of approximately 210 km^2^ (866–2046 m elevation; Figure 1). The traps were moved to new locations approximately every two weeks. The mean ± SD of the distance between two adjacent cameras was 1241 ± 294 m (range of 856–2400 m). The circular size (1.21 km^2^), calculated from the mean distance between two adjacent cameras, was larger than the mean size of the annual home range of Japanese serows [56,57] and that of the summer home range of sika deer [66,67].

The traps were placed at a height of approximately 0.7–1.0 m above the ground, along animal trails, and were not baited. We set the delay period between consecutive events at 1 min and set three burst shots for each event.

### 2.3. Environmental Variables

We used geographic information system software (QGIS version 2.18.16, https://www.npackd.org/p/qgis64/2.18.16, access date 26 November 2021) to evaluate the camera trap detection rates of the Japanese serows and sika deer. The vegetation maps used were at a scale of 1:25,000 and based on the 6–7th Japanese National Survey of the Natural Environment and base map information developed by the Geospatial Information Authority of Japan. Several environmental variables that may influence the habitat use of these two species were used [37,68,69,70,71], including land cover types (deciduous broadleaved forest, coniferous forest, grassland, and farmland), distributions of human land-use (human settlements and road) and water resources, and angle of terrain (hereafter referred to as slope). We obtained the areas of each land cover type and mean slope within buffers of 200 m radii around each camera site, along with distances to the nearest roads, human settlements, and water resources. The resulting buffer area size (12.6 ha) is close in value to the mean home range size of Japanese serows (12.7 ha, computed from Kishimoto and Kawamich [56] and Ochiai and Susaki [57]). We calculated the mean slope within each buffer using points generated every 20 m (1 point per 400 m^2^).

### 2.4. Data Analysis

To assess spatial overlap between Japanese serows and sika deer, we calculated Pianka’s index (α; [72]), where α can range from 0 (no overlap) to 1 (complete overlap), using the photographic capture rates (the number of independent photographs per camera-trap day of sika deer or Japanese serows) at each site. Based on Yasuda [73], consecutive photographs of conspecifics were defined as independent when separated by more than 30 min. Therefore, the same species photographed more than once by the same camera within 30 min was counted as a single event.

The effects of environmental variables on the occurrence of Japanese serows and sika deer at the capture sites were analyzed using a linear regression model. Because there were a large number of sites where the presence of Japanese serows and/or sika deer were not photographed, the following model was assumed to follow a zero-inflated Poisson (ZIP) distribution [74]:

$$\mathrm{ZIP}\left(y|q,\lambda \right)=\{\begin{array}{c} \mathrm{Bernoulli}\;(0|q)+\mathrm{Bernoulli}\;(1|q)\times \mathrm{Poisson}\;(y|\lambda )\;if\;y=0\\ \mathrm{Bernoulli}\;(1|q)\times \mathrm{Poisson}\;(y|\lambda )\;if\;y\geq 1 \end{array}$$

log (*λ*) = *β*_0_ + *β*_1_*z*_1_ + *β*_2_*z*_2_ + *β*_3_*z*_3_ + *β*_4_*z*_4_ + *β*_5_*z*_5_ + *β*_6_*z*_6_ + *β*_7_*z*_7_ + log *T*

where *y* is the number of independent photographs at each site, *q* is the probability indicating the species presence at each site, *λ* is the expected number of independent photographs, *β*_0_ is a constant (intercept), *β*_1–7_ are parameters, *z*_1–7_ are covariates, and *T* is each camera-trap day at each site. The covariates (*z*_1–7_) represent the mean slope (°), distance (km) to the nearest road, distance to the nearest human settlements, distance to the nearest water resource, area (km^2^) of deciduous broadleaved forest, area of coniferous forest, and photographic capture rate of the other study species, respectively.

We created separate models for each species (hereafter referred to as the serow and sika models). Grassland and farmland areas with a variance inflation factor (VIF) larger than 5 were excluded from the analyses to avoid multicollinearity. A VIF exceeding 5 suggests that the model is unstable and has a poor performance [75]. The VIF values of the other variables used in the analyses were <3.73.

Prior to the analysis, all covariates were standardized to have a mean of 0 and a standard deviation of 1. Non-informative priors were used for prior distribution of the parameters. Posterior distributions of all parameters were estimated using the Markov chain Monte Carlo (MCMC) method. Four chains were used for initialization, with 2000 iterations; the first 1000 of which were used for burn-in; the MCMC chains were unthinned. MCMC sampling was considered to be converged when the “R hat” value was <1.1 [76].

To estimate activity patterns and activity levels (the proportion of time that animals spend active) of Japanese serows and sika deer, we fitted non-parametric circular kernel density models [77]. The median time between the first and last consecutive photographs of each species was considered the time of a capture event. We converted local time stamps of independent detections into radian units. We estimated their activity level with 10,000-times smoothed bootstrapping and carried out the randomization test and Wald test to detect differences in activity pattern and activity level between the two species. To determine interspecific overlaps in daily activity, we then estimated the coefficient of temporal overlap (Δ_4_) ranging from 0 (no overlap) to 1 (complete overlap) [78]. We generated 10,000 times smoothed bootstrapping to assess the reliability of the Δ_4_ estimator and to estimate a 95% confidence interval. Statistical significances in interspecific differences of daily activity patterns were assessed using Watson’s two-sample test [79].

We defined spatial (α) and temporal (Δ_4_) overlap indices with ≤50th percentile as “low”, between 50th< and ≤75th percentiles as “moderate”, and >75th percentile as “high” following previous studies [80,81,82]. All statistical analyses were performed in R (v4.0.2; [83]) using “car” package [84,85] for the VIF analysis, the “rstan” package [86,87] for estimation of the posteriors, the Stan code from the “brms” package [88,89] to construct the serow and sika models, the “activity” package [77,90] to estimate activity patterns and activity levels of Japanese serows and sika deer, and the “overlap” [91] and the “CircStats” packages [92] to estimate Δ_4_ and to assess its significance.

## 3. Results

**Figure 2:**
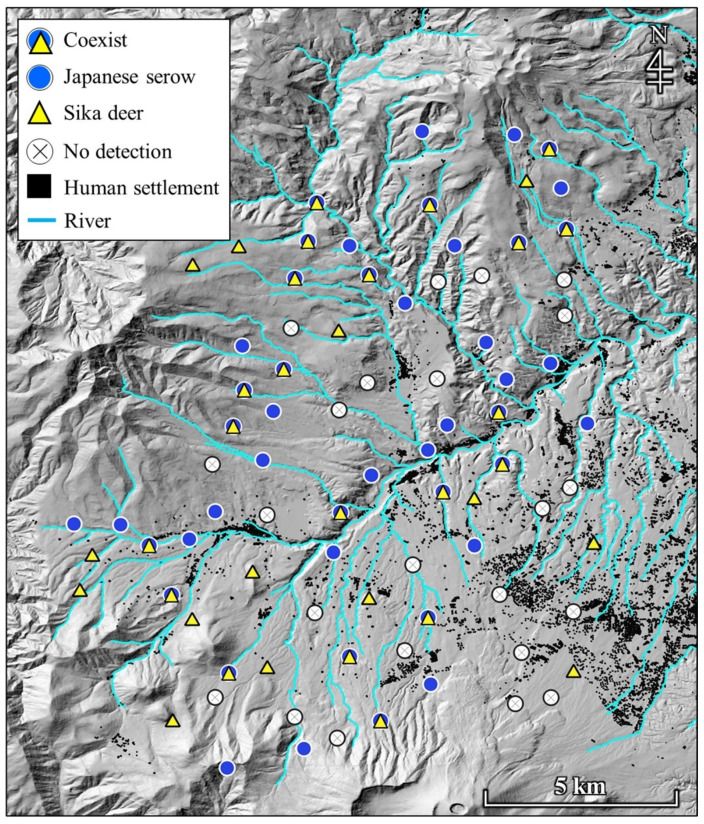
Distribution of Japanese serows and sika deer in Tsumagoi, central Honshu, Japan. Circles and triangles in the figure represent the sites where either Japanese serows or sika deer were photographed by camera traps, respectively. The map was created based on national land and base map information from the Ministry of Land, Infrastructure, Transport and Tourism of Japan.

The cumulative trapping effort over the study period was 1245 camera days. The total number of independent photographs was 96 for the Japanese serows and 88 for the sika deer. Out of 83 camera sites, Japanese serows were photographed at 46 sites and sika deer at 35 sites. The mean ± SD of photographic capture rate (at sites with recorded presence) for each species was 2.09 ± 1.13 for the Japanese serows and 2.51 ± 2.97 for the sika deer. The number of camera sites at which both species, only one species, and neither species were photographed was 21, 39 (25 for the Japanese serows and 14 for the sika deer), and 23 sites, respectively. Both species were photographed throughout the study area (Figure 2). However, we observed low spatial overlap (α = 0.34) between the two species.

**Figure 3:**
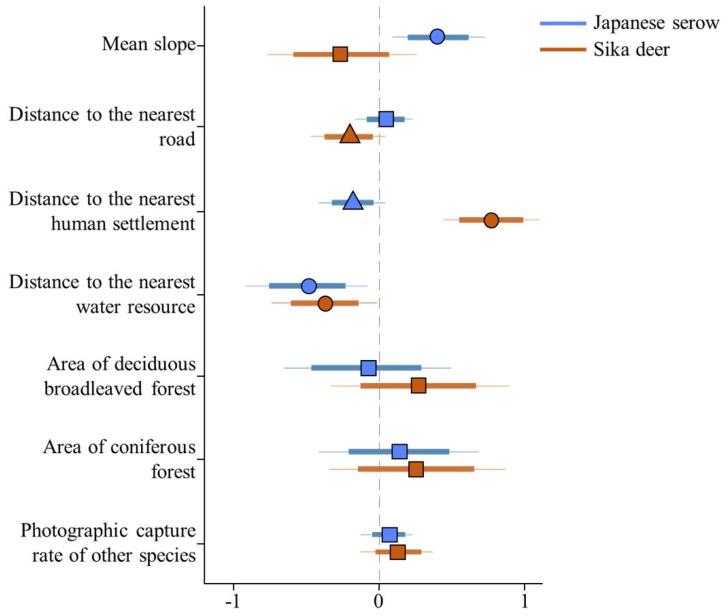
Posterior distribution of the parameters included in the occurrence model of Japanese serows and sika deer in Tsumagoi, central Honshu, Japan. Thin and thick lines represent the 95 and 80% credible intervals (CIs), respectively. Circles, triangles, and squares represent 95, 80 and <80% CI, respectively. Mean slope refers to the mean angle of terrain, and “other species” refers to the other (of the two) study species.

The photographic capture rate of the Japanese serows was significantly positively correlated with the mean slope, and significantly negatively correlated with the distance to the nearest water resource (Figure 3), indicating that this species selected steep slopes and areas close to water resources. The photographic capture rate of the sika deer was significantly positively correlated with the distance to the nearest human settlements, and significantly negatively correlated with the distance to water resources (Figure 3), indicating that this species also selected areas close to water resources, and that they avoided areas close to human settlements. Habitat selection within each species was not affected by the photographic capture rates of the other study species (Figure 3).

**Figure 4:**
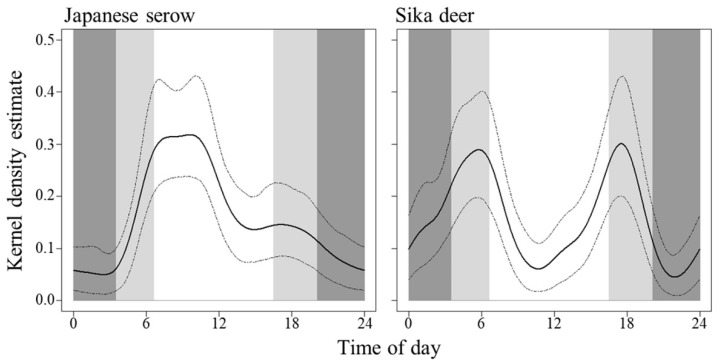
Circular kernel density models showing the overall daily activity patterns of Japanese serow and sika deer in Tsumagoi, central Honshu, Japan. Dashed lines indicate the 95 % confidence intervals. The dark gray-shaded, light gray-shaded, and white areas indicate nighttime, dawn and dusk, and daytime, respectively. The day division was according to Ikeda et al. [52]: dawn (1 h before and after sunrise), dusk (1 h before and after sunset), daytime (from 1 h after sunrise to 1 h before sunset), and nighttime (from 1 h after sunset to 1 h before sunrise). Data on the time of sunrise and sunset in Maebashi (36°23′ N, 139°4′ E), recorded by the National Astronomical Observatory of Japan, were used.

**Figure 5:**
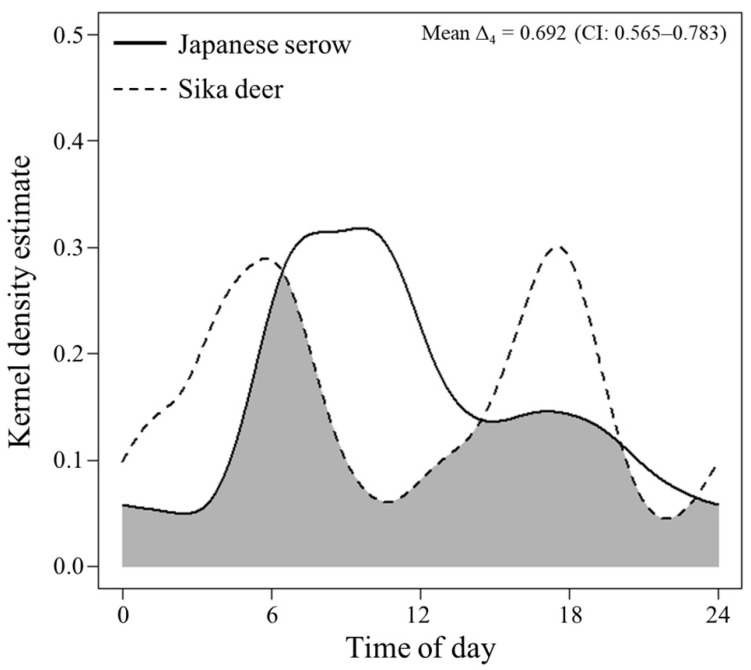
Comparison of daily activity patterns of Japanese serows and sika deer in Tsumagoi, central Honshu, Japan. The grey-shaded area indicates the mean coefficient of overlap, Δ_4_, between both species.

Circular kernel density models indicated that the Japanese serows were more active during the daytime, whereas the sika deer were active at the crepuscule (Figure 4). Their activity patterns were significantly different (*p* < 0.001). We also observed significant temporal partitioning between the two species (mean Δ_4_ = 0.692; 95% confidence interval, 0.565–0.783; *p* < 0.001; Figure 5). The activity levels (the ratio of the areas under and above the curve of the circular probability density function in Figure 4) estimated for the Japanese serows and sika deer were 0.501 (95% confidence interval, 0.349–0.585) and 0.530 (0.355–0.604), respectively, and there was no significant difference (W = 0.105 ± 0.087, *p* = 0.745).

## 4. Discussion

Our results show that the two species exhibited temporal partitioning in their activity patterns, with the Japanese serows and sika deer demonstrating diurnal and crepuscular activity patterns, respectively (Figure 4 and Figure 5). The activity pattern in the sika deer observed in our study is consistent with previous reports of the species in other areas [50,52,53,93,94,95,96]. Although several studies described lower nighttime activity in other populations [50,52,53,94,96], a greater shift toward nocturnality has been apparent in areas with intense human activities [51,54,94,95]. Therefore, the low activity of the sika deer during the daytime probably resulted from the avoidance of humans [52,54,97]. Although little is known concerning the activity patterns of Japanese serows, the observed activity patterns of the Japanese serows were also consistent with a previous report that a radio-collared female was more active during the daytime than nighttime [55]. The Japanese serow has been legally protected since 1955, which might contribute to their diurnal activity. Despite this, and in contrast to the relatively well-known ecology of sika deer, there is a gap in the literature regarding the factors influencing the activity patterns of serow species. The few studies detailing the known activity patterns of other serow species of the genus *Capricornis* reported rather drastic differences. For example, mainland serows (*C*. *sumatraensis*) were observed to be more active at night [98], red serows (*C*. *rubidus*) from the afternoon through to midnight [99], Chinese serows (*C*. *milneedwardsii*) at dawn and dusk [61], and Himalayan serows (*C*. *thar*) more frequently in the morning and night [100]. Chen et al. [99] revealed that the time at which Chinese serows were more active changed from afternoon and midnight in the dry season to between sunrise and noon in the wet season, likely to avoid interference competition with red serows.

The Japanese serows and sika deer were recorded at more than 25% of the same camera sites (Figure 2), but spatial overlap indices between them were low. In addition, although the two species exhibited similar positive selections for habitats close to water resources, the Japanese serows prioritized steep slopes, whereas the sika deer showed a significant avoidance of human settlements (Figure 3). Because neither species’ habitat selection was significantly affected by the photographic capture rate of the other (Figure 3), the observed results could be explained by species-specific requirements rather than by interspecific competition. Our observations that Japanese serows select steep slopes are corroborated by reports describing this habitat selection in other areas where sika deer density was low (<1 individual/km^2^) [101], and where sika deer density was generally high [37,42,43,102,103]. The results indicate that Japanese serows do not select steep slopes to avoid sika deer. In fact, ungulate species with shorter limbs (such as Japanese serows) are specialized for climbing and navigating mountainous habitats, whereas those with longer limbs (such as sika deer) are specialized for speed, to run away from predators in open habitats [104,105]. Moreover, the utilization of steep-sloping terrain such as cliffs is a fundamental predator avoidance strategy in caprids [106]. In a study investigating this specific aspect of Japanese serow behavior, Takada et al. [107] suggested that their selection for steep and low-visibility habitats can be attributed to security. Thus, predator avoidance mechanisms could be a major factor influencing the difference in the observed selection for steep slopes between the Japanese serows and sika deer.

In contrast to Japanese serows, the most significant factor determining sika deer occurrence was (a disinclined) proximity to human settlements (Figure 3). Human disturbance likely influences the behavior, habitat use, and activity patterns of sika deer [52,54,97,108,109,110,111]. As deer species are extremely sensitive to human disturbance [47], the negative selection for human proximity may be a consequence of spatial human avoidance of sika deer. However, Japanese serows appear less sensitive to human presence, as the recorded abundance of Japanese serows tended to be high in areas close to human settlements (Figure 3). The difference between the sika deer and Japanese serows regarding the avoidance of human settlements could be a consequence of the difference in hunting pressure of each species; specifically, the sika deer is a game species, whereas the Japanese serow is a legally protected species.

We observed that the Japanese serows and sika deer selected areas close to water resources (Figure 3). Similarly, Ishida et al. [101] revealed that both these ungulate species were typically observed around valley-like terrain. Water is a critical resource for Cervidae and Bovidae in arid regions [112,113,114,115]. Although the climate in Japan is humid, the drinking water behaviors of the Japanese serows and sika deer observed in several areas [116,117,118] suggest that water is an important factor determining the behavior of these ungulates. To adequately assess the importance of water resources for Japanese serows and sika deer, systematic surveys for water use by these ungulates need to be conducted.

Japanese serows are smaller than sika deer (29.0–56.5 kg [119] versus 37.6–100.0 kg [120]), and larger species are typically superior to smaller ones [46]. Indeed, Japanese serows tend to avoid sika deer when they encounter each other, whereas sika deer likely ignore the presence of Japanese serows [121]. Although the Japanese serows and sika deer exhibited similar positive selection for water resources (Figure 3), the temporal partitioning observed between the two species reduced their encounter rates, minimizing possible interference competition between them. Similar partitioning mechanisms are known to occur not only within other sympatric herbivore communities [49,61,99,100,122] but also within sympatric carnivore communities [21,123]. However, Koganezawa [36] revealed that population densities of Japanese serows decreased in areas where sika deer density exceeded 25 individuals/km^2^; however, their densities were not significantly different in an area where sika deer density was <10 individuals/km^2^. As the population density of sika deer increases, the encounter rate between the two species is also expected to increase, due to a moderate temporal overlap between them (Figure 5), which may influence the population levels of Japanese serows.

Although snare traps have been widely used for sika deer management, Japanese serows have typically been unintentionally captured, and many individuals have been injured in the process, sometimes fatally [59,124]. Our study sheds light on possible measures that can be adopted to minimize the risks of these accidents. The Japanese serows and sika deer selected areas close to water resources (Figure 3). The Japanese serows also selected steep slopes, whereas sika deer did not show any preference for slopes (Figure 3). In addition, both ungulates exhibited opposing tendencies with regard to the proximity of human settlements, with the Japanese serows tending to select areas close to human settlements, whereas sika deer selected areas far from human settlements (Figure 3). While trapping near human settlements reduces trapping effort from an accessibility perspective, this may increase the unintentional capture of Japanese serows. Thus, to reduce the unintentional capture of Japanese serows, sika deer trapping efforts near valleys should be minimized and instead focused on areas with gentler slopes away from human settlements.

## 5. Conclusions

The results of this study support our hypotheses that Japanese serows and sika deer exhibit spatial as well as temporal partitioning. This partitioning likely reduces the encounter rates between the two species, minimizing possible interference competition between them. However, spatial and temporal overlaps between Japanese serows and sika deer are likely to increase with the increase in sika deer density, which may result in a decline in the population density or a niche shift of Japanese serows with smaller body size. To better understand the factors influencing declines in the population of Japanese serows, more research needs to be conducted in areas with varied sika deer densities. Our study also identifies the following possible avenues to reduce the unintentional capture of the threatened Japanese serow in trapping efforts aimed at sika deer: trapping should be focused on areas with gentler slopes, away from valleys and human settlements.

## Data Availability

The data presented in this study are available from the corresponding author on reasonable request.
